# Supplementary material for: Differences in the pupillary responses to evening light between children and adolescents
Source: J Physiol Anthropol. 2024 Jul 3;43:16. doi: 10.1186/s40101-024-00363-6 (PMC11221120; doi:10.1186/s40101-024-00363-6)
Supplement: Supplementary file 1 — Supplementary Material 1. [file 40101_2024_363_MOESM1_ESM.docx]

**SUPPLEMENTAL MATERIALS**

**Fig. S1. Visible light transmission through dark glasses.**

**Fig. S2. Spectra of experimental light conditions.**

**Table S1**. Means and standard deviations of each pupillary feature broken down by age group and experimental light condition.

| **Variable** | **Children** | | **Adolescents** | |
| --- | --- | --- | --- | --- |
|  | **Red** | **Blue** | **Red** | **Blue** |
| **Phasic Constriction (%)** | 21.04 (3.84) | 21.62 (5.62) | 16.86 (4.09) | 18.13 (4.12) |
| **Constriction Latency (s)** | 0.33 (0.06) | 0.33 (0.07) | 0.39 (0.06) | 0.37 (0.06) |
| **Max Constriction (%)** | 55.72 (4.56) | 57.85 (4.74) | 50.87 (5.02) | 53.62 (4.07) |
| **Sustained Slope (%/s)** | -1.13 (0.81) | -0.59 (0.53) | -1.00 (0.62) | -0.24 (0.41) |
| **PIPR (%)** | 15.31 (7.99) | 14.89 (4.82) | 11.39 (4.59) | 13.48 (4.43) |
| **AUC (%.s)** | 213.30 (70.91) | 218.80 (39.11) | 179.83 (54.48) | 205.55 (45.83) |
